# Supplementary material for: Centromeres are maintained by fastening CENP-A to DNA and directing an arginine anchor-dependent nucleosome transition
Source: Nat Commun. 2017 Jun 9;8:15775. doi: 10.1038/ncomms15775 (PMC5472775; doi:10.1038/ncomms15775)
Supplement: Supplementary Information — Supplementary Figures and Supplementary References [file ncomms15775-s1.pdf]

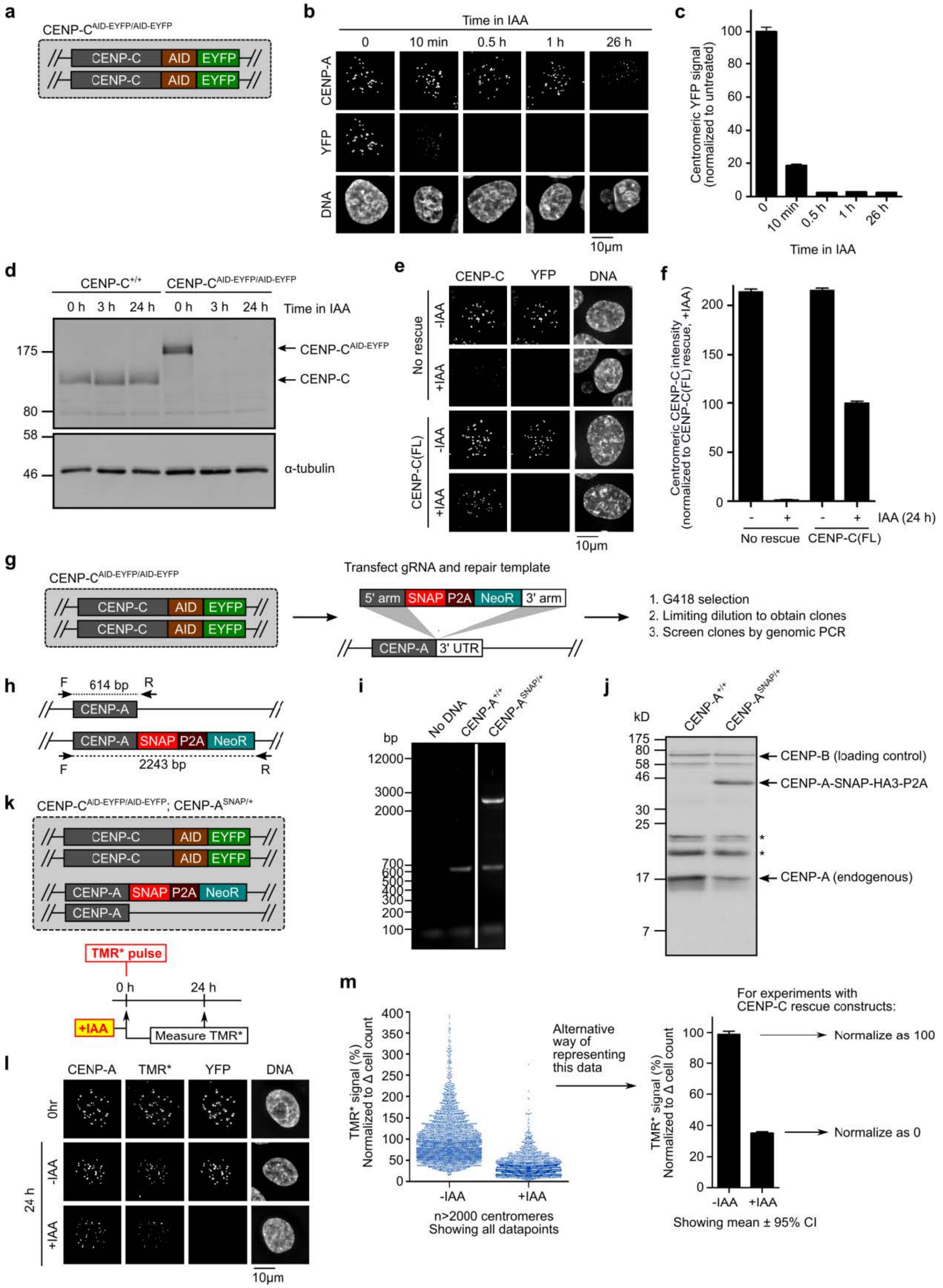

a
CENP-C AID-EYFP/AID-EYFP
CENP-C AID EYFP
b
Time in IAA
0 10 min 0.5 h 1 h 26 h
CENP-A
YFP
DNA
10µm
c
Centromeric YFP signal (normalized to untreated)
Time in IAA
d
CENP-C+/+
CENP-C AID-EYFP/AID-EYFP
0 h 3 h 24 h 0 h 3 h 24 h Time in IAA
CENP-C AID-EYFP
CENP-C
α-tubulin
e
CENP-C YFP DNA
No rescue
CENP-C(FL)
-IAA
+IAA
f
Centromeric CENP-C intensity (normalized to CENP-C(FL) rescue, +IAA)
IAA (24 h)
No rescue CENP-C(FL)
g
Transfect gRNA and repair template
5' arm SNAP P2A NeoR 3' arm
CENP-A 3' UTR
1. G418 selection
2. Limiting dilution to obtain clones
3. Screen clones by genomic PCR
h
614 bp
2243 bp
CENP-A SNAP P2A NeoR
i
No DNA CENP-A+/+ CENP-ASNAP/+
bp
j
kD
CENP-A+/+ CENP-ASNAP/+
CENP-B (loading control)
CENP-A-SNAP-HA3-P2A
CENP-A (endogenous)
k
CENP-C AID-EYFP/AID-EYFP; CENP-A SNAP/+
TMR* pulse
0 h 24 h
+IAA
Measure TMR*
l
CENP-A TMR* YFP DNA
0hr
24 h
-IAA
+IAA
10µm
m
TMR* signal (%) Normalized to Δ cell count
-IAA +IAA
n>2000 centromeres
Showing all datapoints
Alternative way of representing this data
For experiments with CENP-C rescue constructs:
Normalize as 100
Normalize as 0
Showing mean ± 95% CI

Supplementary Figure 1. Compromised retention of centromeric CENP-A nucleosomes upon rapid auxin-induced degradation of CENP-C.

(a) Schematic of CENP-$C^{AID\text{-}EYFP/AID\text{-}EYFP}$ cells.

(b) Timecourse of CENP-C-AID-EYFP signal after various lengths of IAA treatment.

(c) Quantitation of b. Mean ± 95% confidence interval (n>2000 centromeres in all cases).

(d) Immunoblot of CENP-$C^{AID\text{-}EYFP/AID\text{-}EYFP}$ cells using anti-CENP-C and anti-α-tubulin after various lengths of IAA treatment.

(e) CENP-$C^{AID\text{-}EYFP/AID\text{-}EYFP}$ cells expressing full-length, untagged CENP-C rescue construct at the unique FRT site. Prior to IAA treatment, anti-CENP-C antibody detects both the rescue construct and the CENP-C-AID-EYFP at the endogenous gene locus. After IAA treatment, the AID-tagged CENP-C is depleted (as verified by the loss of YFP signal), and anti-CENP-C antibody exclusively detects the rescue construct.

(f) Quantitation of e. Since both CENP-C-AID-EYFP and the rescue construct are present in the cell prior to IAA addition, there is an expected drop (~2-fold) in centromeric CENP-C signal upon rapid removal of the AID-tagged CENP-C. Mean ± 95% confidence interval (n>2000 centromeres in all cases).

(g) Schematic for SNAP-tagging CENP-A at its endogenous locus by CRISPR/Cas, in CENP-$C^{AID\text{-}EYFP/AID\text{-}EYFP}$ cells.

(h) Schematic for screening clones by genomic PCR. Incorporation of the repair template containing SNAP is expected to result in a 2.2 kb PCR product.

(i) PCR with genomic DNA extracted from parental cells and a heterozygous clone in which one allele in which CENP-A is SNAP-tagged by CRISPR.

(j) Verification of presence of SNAP-tagged CENP-A by immunoblot with anti-centromere antibodies (ACA). In addition to CENP-A, ACA also recognizes CENP-B, which here serves as a loading control. The heterozygous clone has both endogenous CENP-A as well as SNAP-tagged CENP-A.

(k) Schematic of the clone used for all TMR* experiments in this study (CENP-$C^{AID\text{-}EYFP/AID\text{-}EYFP}$ cells with CENP-A that is SNAP-tagged at its endogenous locus), and schematic of pulse-chase experiment to measure CENP-A retention at the centromere after complete depletion of CENP-C.

(l) Representative images from experiment diagrammed in k.

(m) Left: Quantitation of panel l, shown with all datapoints (n>2000 centromeres in all cases), and displayed as in our prior study[1]. Right: Plotting these same data as a bar graph showing mean ± 95% confidence interval[2]. For TMR* experiments with CENP-C rescue constructs (Figs. 1g-i, 3d,e, and 7d-f), the value of the -IAA condition is normalized as 100%, and the value of the +IAA condition is normalized as 0%.

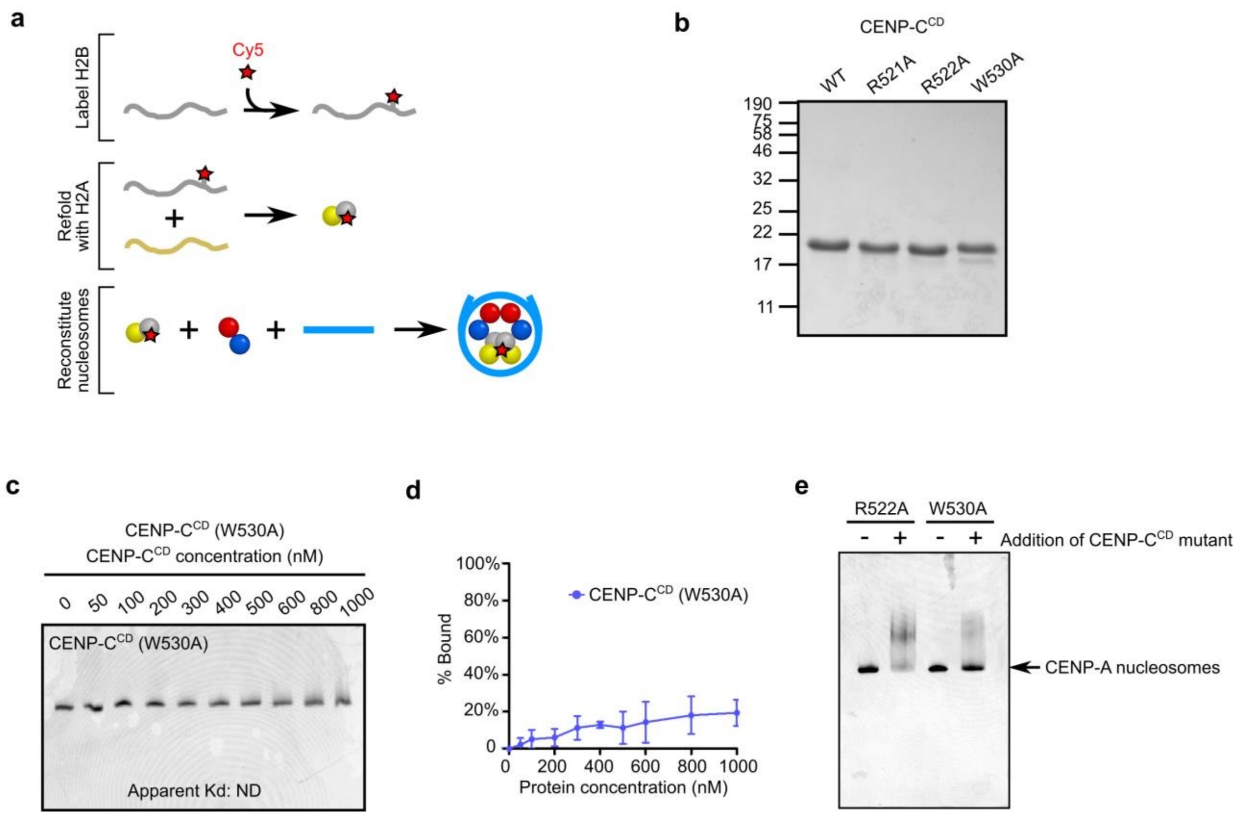

Supplementary Figure 2. CENP-C^{CD}(W530A) fails to bind to CENP-A nucleosomes.
(a) Schematic for labeling H2B K120C with Cy5, subsequent refolding with H2A to form histone dimers, and reconstitution into nucleosomes.
(b) SDS-PAGE gels stained with Coomassie Blue of CENP-C^{CD} WT and mutant proteins used for binding assays shown in this figure and Fig. 2.
(c) Representative native PAGE analysis of CENP-A NCPs harboring Cy5-labeled histone H2B that have been incubated with the indicated concentrations of CENP-C^{CD}(W530A). Only low level of binding to the nucleosome was observed for the W530A mutant.
(d) Quantitation of three independent experiments (values shown are mean ± SD) performed as in panel c. Note that for some data points, the error bars are too small to be visible in the graph. The apparent K_d for W530A was not determined (ND) because of insufficient binding.
(e) The W530A mutant does not assemble with CENP-A nucleosomes to an extent that would make an HXMS experiment useful or interpretable. CENP-C^{CD} mutants incubated with CENP-A nucleosomes containing Cy5-H2B. CENP-C^{CD} (R522A) can form a complex with CENP-A nucleosomes (under the same conditions as those used to assemble nucleosomes for the HXMS experiments in Fig. 2), while at this concentration, CENP-C^{CD} (W530A) displays very little binding to CENP-A nucleosomes (note that the majority of the

nucleosomes in the presence of W530A are unbound from the mutant CENP-C protein). The high proportion of unbound nucleosomes would dominate the HXMS experiment and would not be capable for making a useful comparison to wild type or R522A versions of CENP-C.

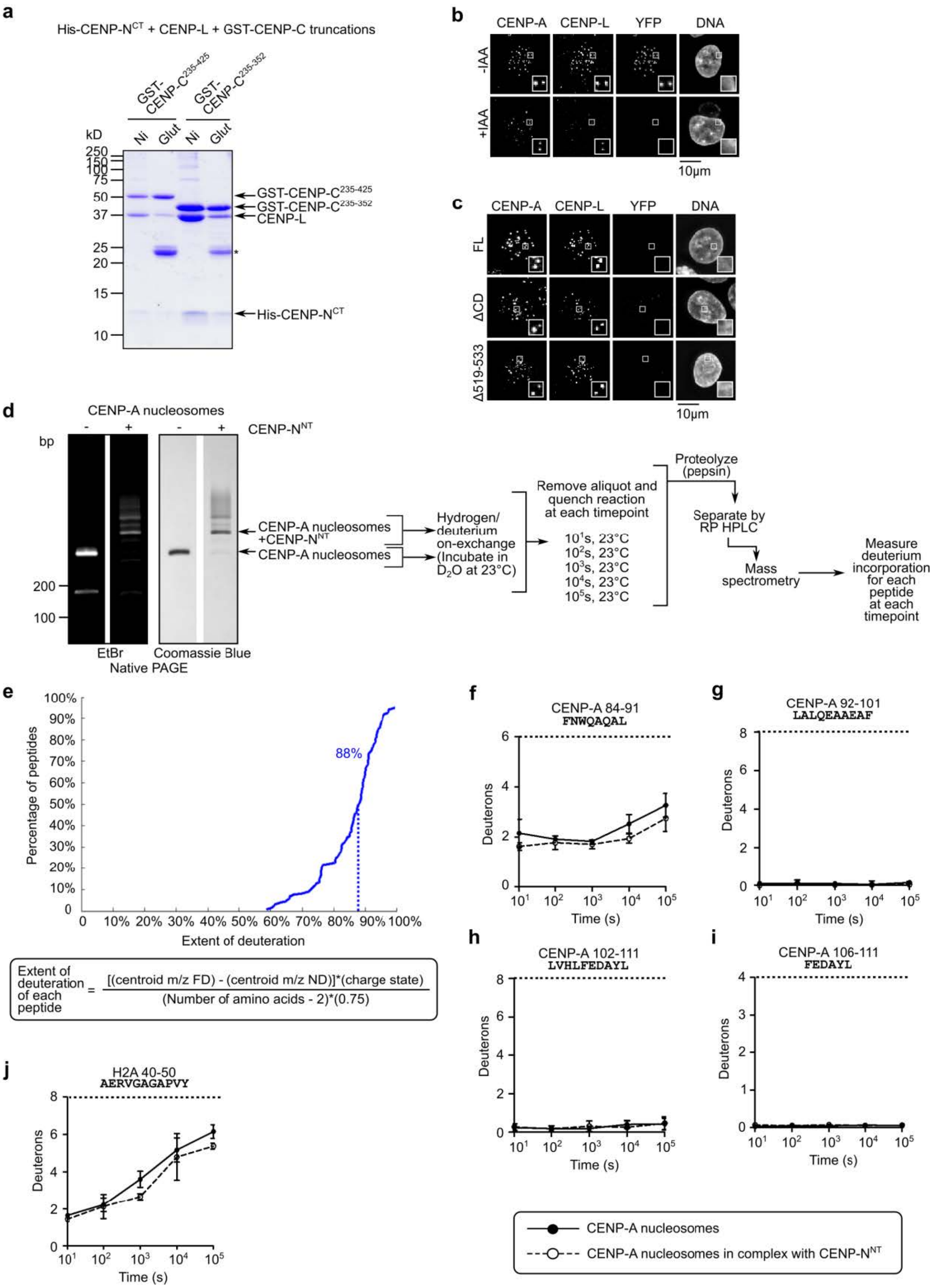
a
His-CENP-N^{CT} + CENP-L + GST-CENP-C truncations
GST-CENP-C$^{235-425}$
GST-CENP-C$^{235-352}$
kD
Ni
Glut
Ni
Glut
250
150
100
75
50
37
25
20
15
10
GST-CENP-C$^{235-425}$
GST-CENP-C$^{235-352}$
CENP-L
*
His-CENP-N^{CT}
b
CENP-A
CENP-L
YFP
DNA
-IAA
+IAA
10μm
c
CENP-A
CENP-L
YFP
DNA
FL
ΔCD
Δ519-533
10μm
d
CENP-A nucleosomes
CENP-N^{NT}
bp
200
100
EtBr
Coomassie Blue
Native PAGE
CENP-A nucleosomes +CENP-N^{NT}
CENP-A nucleosomes
Hydrogen/ deuterium on-exchange (Incubate in D_2O at 23°C)
Remove aliquot and quench reaction at each timepoint
10^1s, 23°C
10^2s, 23°C
10^3s, 23°C
10^4s, 23°C
10^5s, 23°C
Proteolyze (pepsin)
Separate by RP HPLC
Mass spectrometry
Measure deuterium incorporation for each peptide at each timepoint
e
Percentage of peptides
88%
Extent of deuteration
Extent of deuteration of each peptide = [(centroid m/z FD) - (centroid m/z ND)]*(charge state) / (Number of amino acids - 2)*(0.75)
f
CENP-A 84-91
FNWQAQAL
g
CENP-A 92-101
LALQEAAEAF
h
CENP-A 102-111
LVHLFEDAYL
i
CENP-A 106-111
FEDAYL
j
H2A 40-50
AERVGAGAPVY
Deuterons
Time (s)
CENP-A nucleosomes
CENP-A nucleosomes in complex with CENP-N^{NT}

Supplementary Figure 3. CENP-L/N^{CT} binds CENP-C$^{235-352}$, and CENP-N^{NT} binds the CENP-A nucleosome surface bulge.

(a) SDS-PAGE showing co-purification performed as described[3] of fragments of GST-CENP-C with CENP-L/His-CENP-N^{CT}, demonstrating that CENP-C$^{235-352}$ is sufficient for the interaction with CENP-L/N^{CT}.

(b) Representative images showing localization of CENP-L/N in CENP-C$^{AID-EYFP/AID-EYFP}$ cells before and after 24 h of IAA treatment, assessed using anti-CENP-L[3]. See quantitation in Fig. 4b.

(c) Representative images showing localization of CENP-L/N in CENP-C$^{AID-EYFP/AID-EYFP}$ cells constitutively expressing the rescue constructs CENP-C(FL), CENP-C(ΔCD), or CENP-C(Δ519-533), after 24 h of IAA treatment, assessed using anti-CENP-L[3] . See quantitation in Fig. 4c.

(d) CENP-A NCPs alone and in complex with CENP-N^{NT} as assessed by native PAGE stained with EtBr or Coomassie Blue, and schematic for HXMS experiment.

(e) Evidence that our HXMS experiments have minimal back-exchange. Cumulative distribution curve of a representative fully deuterated (FD) sample (see the Methods for a description of how FD samples are prepared), showing the extent of deuteration of all peptides compared to the theoretical maximum amount of deuteration of each peptide (i.e., if every amide proton were exchanged for a deuteron). The median deuteration was ~88% for the FD sample, therefore the back-exchange after the quench step was only ~12%, which is well within the optimal range (better than most published HXMS experiments[4]).

(f-i) Representative peptides spanning the α2 helix of CENP-A. We note that our data do not exclude that the binding site might extend to adjacent surface residues on the N-terminal portion of α2 helix[5,6] that also lies within the CATD. Even prior to CENP-N^{NT} binding, this particular region (CENP-A a.a. ~85-111) of the α2 helix of CENP-A undergoes HX too slowly (>12 days[7]) for us to detect. All peptides are plotted at every time point as mean ± SD from triplicate experiments. At every other location of the folded core of the octameric CENP-A nucleosome, however, HX is fast enough that we could have measured changes if imparted by CENP-N^{NT}, but none were observed (See Fig. 4d). Note that for some data points, the error bars are too small to be visible in the graph.

(j) A peptide of H2A a.a. 40-50, showing mild protection upon binding to CENP-N.

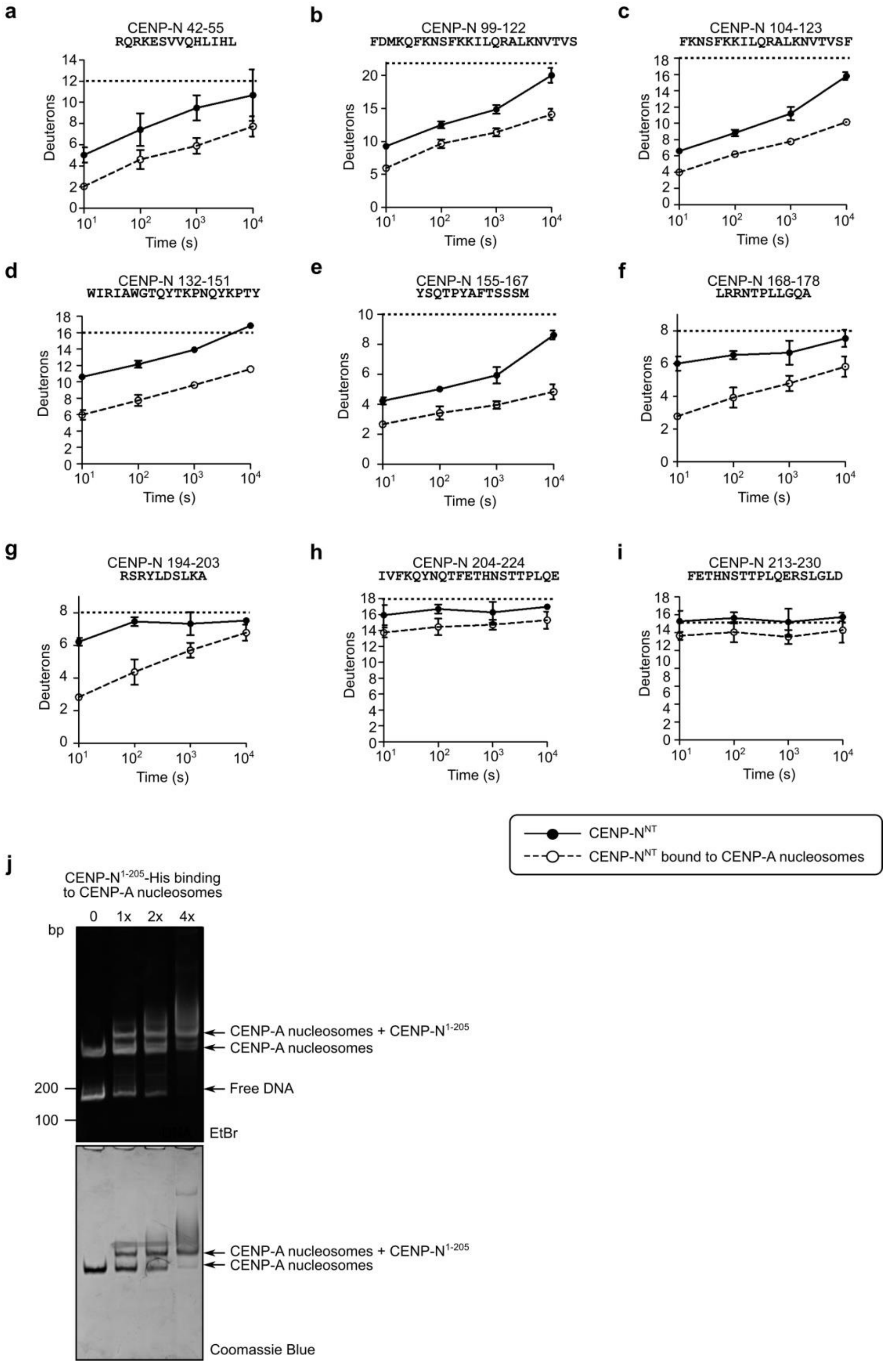

a
CENP-N 42-55
RQRKESVVQHLIHL
b
CENP-N 99-122
FDMKQFKNSFKKILQRALKNVTVS
c
CENP-N 104-123
FKNSFKKILQRALKNVTVSF
d
CENP-N 132-151
WIRIAWGTQYTKPNQYKPTY
e
CENP-N 155-167
YSQTPYAFTSSSM
f
CENP-N 168-178
LRRNTPLLGQA
g
CENP-N 194-203
RSRYLDSLKA
h
CENP-N 204-224
IVFKQYNQTFETHNSTTPLQE
i
CENP-N 213-230
FETHNSTTPLQERSLGLD
Deuterons
Time (s)
CENP-N^NT
CENP-N^NT bound to CENP-A nucleosomes
j
CENP-N^1-205-His binding to CENP-A nucleosomes
0
1x
2x
4x
bp
200
100
CENP-A nucleosomes + CENP-N^1-205
CENP-A nucleosomes
Free DNA
EtBr
CENP-A nucleosomes + CENP-N^1-205
CENP-A nucleosomes
Coomassie Blue

Supplementary Figure 4. The N-terminal 205 amino acids of CENP-N constitute its minimal nucleosome-binding domain.

(a-i) Representative peptides spanning various regions of CENP-N^{NT}. CENP-N^{NT} experiences substantial protection from HX across its entire first ~200 amino acids (a-g), indicating that it consists of a folded domain that becomes globally rigidified upon binding to CENP-A NCPs. The residues 206-230 (h,i) reach full deuteration even at the earliest timepoint, and show no difference upon binding to CENP-A NCPs, which indicates that this is a disordered region potentially dispensable for binding to CENP-A NCPs. The maximum number of deuterons possible to measure by HXMS for each peptide is shown by the dotted line. All peptides are plotted at every time point as mean ± SD from triplicate experiments. Note that for some data points, the error bars are too small to be visible in the graph.

(j) Guided by HXMS data (a-i), we further truncated CENP-N^{NT} into just its first 205 amino acids. Native PAGE shows CENP-A NCPs with increasing molar ratios of CENP-$N^{1\text{-}205}$, stained either with EtBr or Coomassie Blue. As expected, CENP-$N^{1\text{-}205}$ is sufficient for binding to CENP-A NCPs, supporting the notion that this is the minimal nucleosome-binding domain on CENP-N.

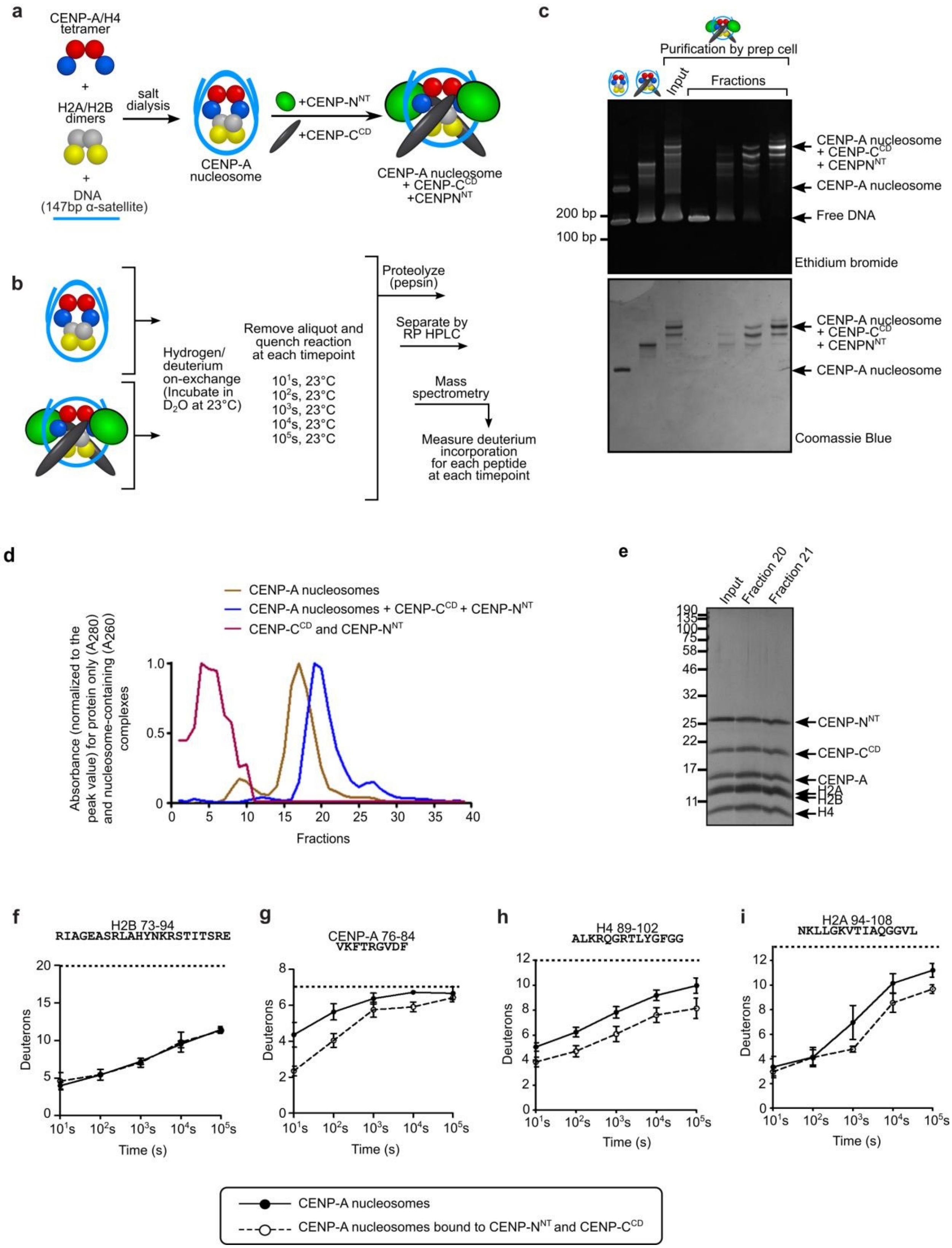

a
CENP-A/H4 tetramer
H2A/H2B dimers
DNA (147bp α-satellite)
salt dialysis
CENP-A nucleosome
+CENP-N^NT
+CENP-C^CD
CENP-A nucleosome + CENP-C^CD +CENPN^NT
b
Hydrogen/ deuterium on-exchange (Incubate in D2O at 23°C)
Remove aliquot and quench reaction at each timepoint
10^1s, 23°C
10^2s, 23°C
10^3s, 23°C
10^4s, 23°C
10^5s, 23°C
Proteolyze (pepsin)
Separate by RP HPLC
Mass spectrometry
Measure deuterium incorporation for each peptide at each timepoint
c
Purification by prep cell
Input
Fractions
200 bp
100 bp
CENP-A nucleosome + CENP-C^CD + CENPN^NT
CENP-A nucleosome
Free DNA
Ethidium bromide
Coomassie Blue
d
CENP-A nucleosomes
CENP-A nucleosomes + CENP-C^CD + CENP-N^NT
CENP-C^CD and CENP-N^NT
Absorbance (normalized to the peak value) for protein only (A280) and nucleosome-containing (A260) complexes
Fractions
e
Input
Fraction 20
Fraction 21
CENP-N^NT
CENP-C^CD
CENP-A
H2A
H2B
H4
f
H2B 73-94
RIAGEASRLAHYNKRSTITSRE
g
CENP-A 76-84
VKFTRGVDF
h
H4 89-102
ALKRQGRTLYGFGG
i
H2A 94-108
NKLLGKVTIAQGGVL
Deuterons
Time (s)
CENP-A nucleosomes
CENP-A nucleosomes bound to CENP-N^NT and CENP-C^CD

Supplementary Figure 5. CENP-A NCPs in complex with both CENP-N^{NT} and CENP-C^{CD} experience additive HX protection.

(a,b) Experimental scheme for HXMS of the CENP-A NCP in complex with both CENP-N^{NT} and CENP-C^{CD}.

(c) Purification of the complex of CENP-A nucleosomes bound to CENP-C^{CD} and CENP-N^{NT} (the CCNC) by preparative native PAGE ("Prep Cell"). Fractions are collected as they are eluted out of the bottom of the gel. The native PAGE is stained both by ethidium bromide (For DNA) and Coomassie Blue (for protein).

(d) Sucrose gradient elution profiles of CENP-A nucleosomes, CENP-A nucleosomes in complex with CENP-C^{CD} and CENP-N^{NT}, and the CENP-C^{CD} and CENP-N^{NT} proteins alone. Complexes were subject to a linear 5-30 % sucrose gradient with centrifugation at 35,000 rpm for 13 hr at 4°C. The samples were fractionated from top to bottom, and each fraction was analyzed for absorbance at 280 nm (for nucleosome and complex) or 260 nm (for CENP-C and CENP-N proteins alone). The absorbance values are plotted, with the highest value in each run normalized to 1.0.

(e) SDS-PAGE gels stained with Coomassie-Blue, showing input and peak fractions of CENP-A nucleosomes bound to CENP-C^{CD} and CENP-N^{NT} (the CCNC).

(f-i) Representative peptides within the CCNC. (f) A histone peptide that shows no difference between CENP-A nucleosomes vs. the CCNC. (g-i) Histone peptides spanning the CENP-A surface bulge (g), and the β-sheet region between histone H4 and H2A (h,i) over the timecourse. The maximum number of deuterons possible to measure by HXMS for each peptide is shown by the dotted line. All peptides are plotted at every time point as mean ± SD from triplicate experiments. Note that for some data points, the error bars are too small to be visible in the graph.

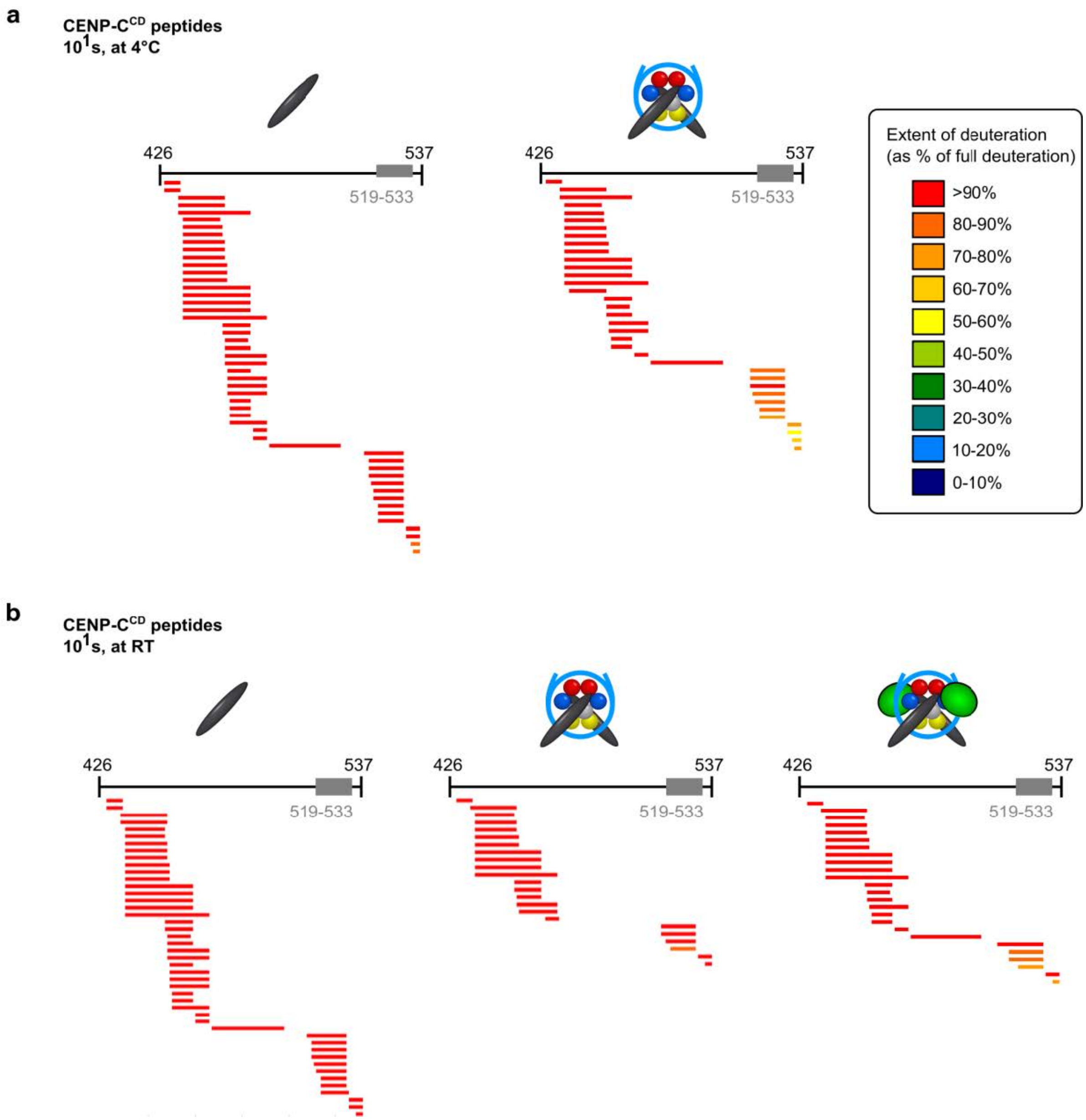

Supplementary Figure 6. CENP-C^{CD} lacks detectable secondary structure and binds the histone surface of CENP-A nucleosomes with residues ~515-537.

(a) CENP-C^{CD} peptides alone vs. when in complex with CENP-A nucleosomes, at the 10^1 s, 4°C timepoint (which is the earliest timepoint we can test, and is equivalent to 10^0 s at room temperature [RT]). This shows that CENP-C^{CD}, when alone, is disordered and is mostly completely exchanged even at this earliest timepoint. CENP-C^{CD} when in complex with CENP-A nucleosomes shows some mild protection in the a.a. 513-537 region, which encompasses the region that contacts CENP-A nucleosomes (~a.a. 519-533). Each

horizontal bar represents an individual peptide from CENP-C^{CD} alone or in complex with CENP-A nucleosomes and is color-coded for percent deuteration.

(b) CENP-C^{CD} peptides alone versus when in complex with CENP-A nucleosomes versus when part of the CCNC, at the 10^1 s (RT) timepoint. Again, some mild protection is seen in the a.a. 513-537 region when CENP-C^{CD} is bound to CENP-A nucleosomes or part of the CCNC, but CENP-C^{CD} still overall lacks secondary structure in three instances. Thus, because protection is so minor at such an early timepoint (10 s), the presence of CENP-N does not cause the gain of secondary structure or any other change that would otherwise lead to strong protection from HX.

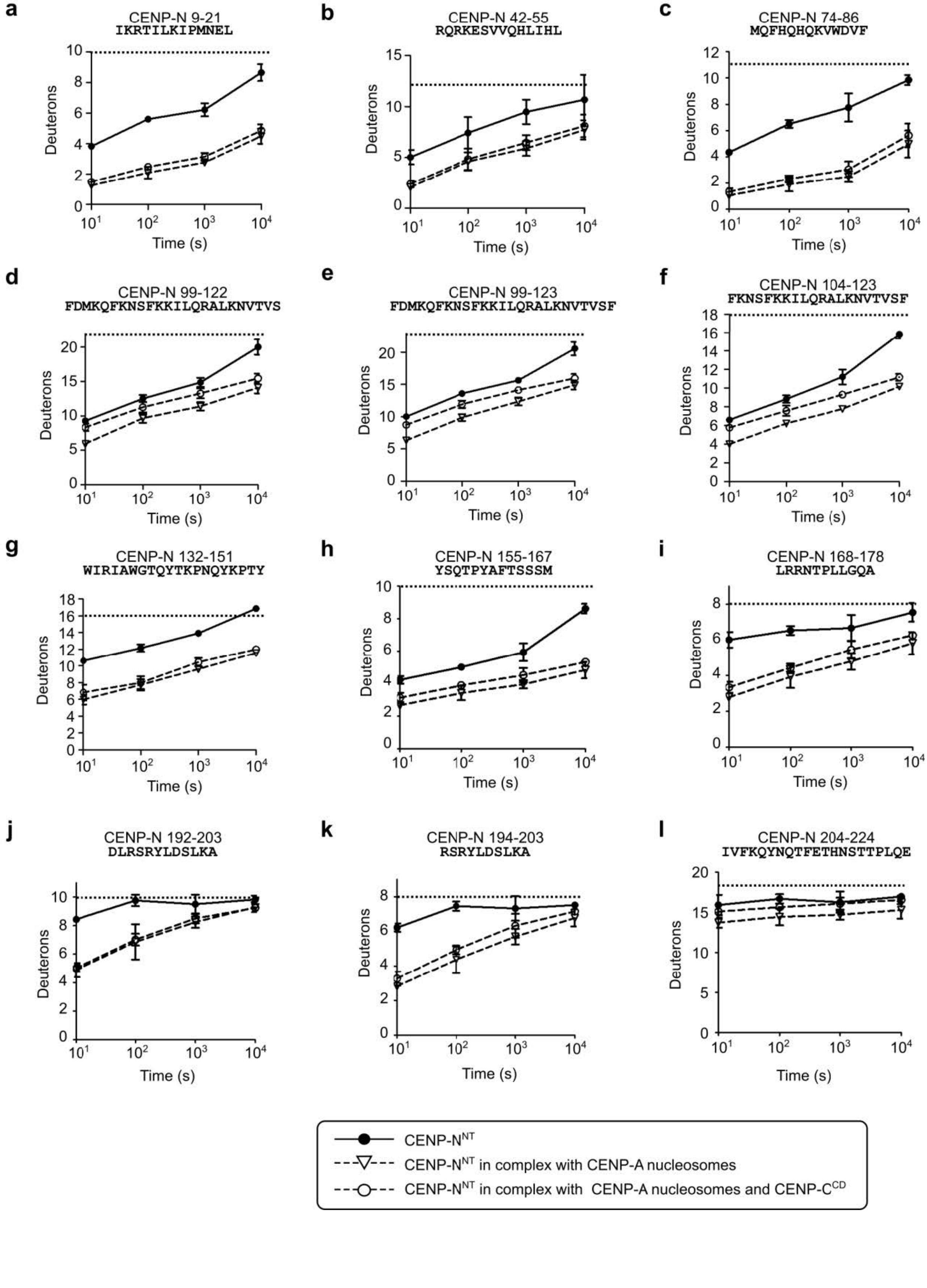
a
CENP-N 9-21
IKRTILKIPMNEL
b
CENP-N 42-55
RQRKESVVQHLIHL
c
CENP-N 74-86
MQFHQHQKVWDVF
d
CENP-N 99-122
FDMKQFKNSFKKILQRALKNVTVS
e
CENP-N 99-123
FDMKQFKNSFKKILQRALKNVTVSF
f
CENP-N 104-123
FKNSFKKILQRALKNVTVSF
g
CENP-N 132-151
WIRIAWGTQYTKPNQYKPTY
h
CENP-N 155-167
YSQTPYAFTSSSM
i
CENP-N 168-178
LRRNTPLLGQA
j
CENP-N 192-203
DLRSRYLDSLKA
k
CENP-N 194-203
RSRYLDSLKA
l
CENP-N 204-224
IVFKQYNQTFETHNSTTPLQE
Deuterons
Time (s)
CENP-N^NT
CENP-N^NT in complex with CENP-A nucleosomes
CENP-N^NT in complex with CENP-A nucleosomes and CENP-C^CD

Supplementary Figure 7. The HXMS behavior of CENP-N^{NT} in the presence and absence of CENP-C^{CD}.

(a-l) Representative peptides of CENP-N^{NT} across the timecourse, either unbound or bound to CENP-A NCPs (in the presence or absence of CENP-C^{CD}). The maximum number of deuterons possible to measure by HXMS for each peptide is shown by the dotted line. All peptides are plotted at every time point as mean ± SD from triplicate experiments. Note that for some data points, the error bars are too small to be visible in the graph. Across *most* of the folded nucleosome-binding domain of CENP-N^{NT}, the massive slowing of HX is similar in the presence or absence of CENP-C^{CD} (a-c, g-k). Within a region that maps unambiguously to a.a. 99-122, however, CENP-N only exhibits substantial HX protection when bound alone to the CENP-A NCP (d-f). This suggests that a very local region (i.e. between a.a. 99-122) has a structural change in the presence of CENP-C that leads to the change in HX behavior we observe. The region C-terminal of a.a. 206 is disordered regardless of whether CENP-N^{NT} is bound to the NCP (l, also see Supplementary Fig. 4).

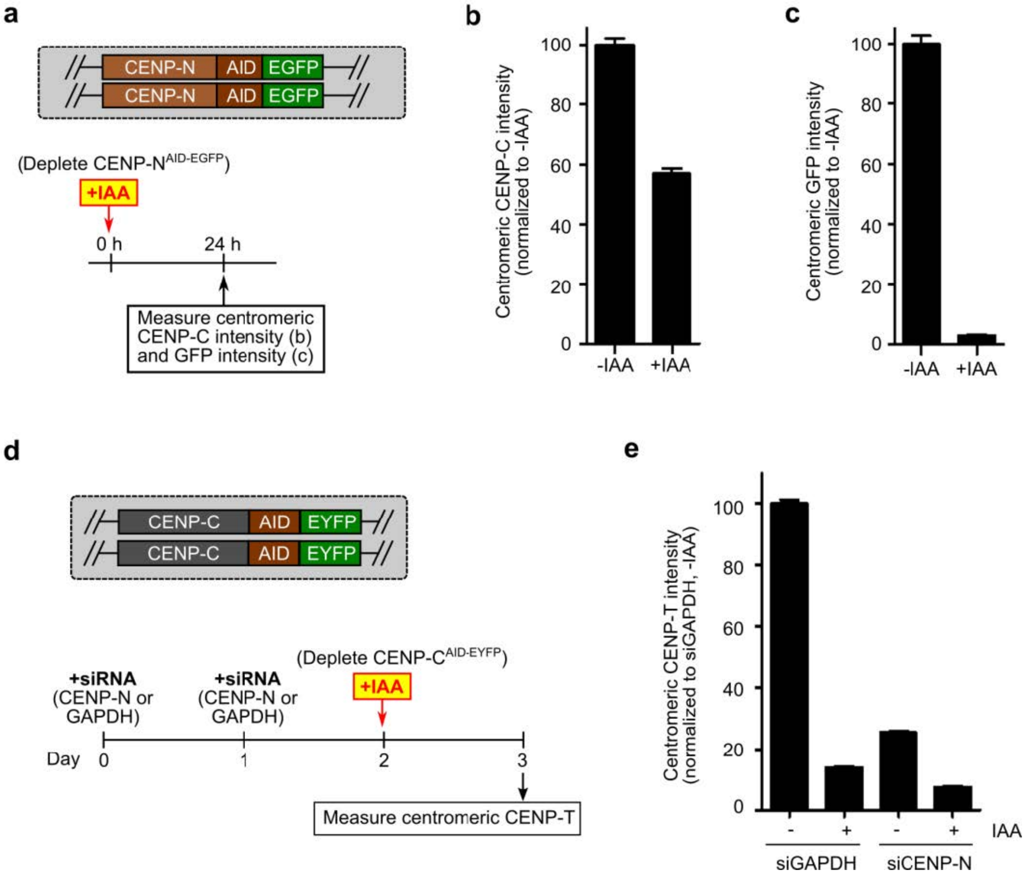

Supplementary Figure 8. Effect of CENP-N depletion on centromeric CENP-C and CENP-T levels.

(a) AID-tagged CENP-N was depleted by 24 h of IAA in CENP-N$^{AID-EGFP/AID-EGFP}$ cells, then assessed for centromeric CENP-C localization, and disappearance of CENP-N-AID-EGFP.

(b) Quantitation of centromeric CENP-C intensity before and after 24 h IAA treatment. Mean ± 95% confidence interval (n>2000 centromeres in all cases).

(c) Quantitation of centromeric CENP-N-AID-EGFP intensity before and after 24 h IAA treatment. Mean ± 95% confidence interval (n>2000 centromeres in all cases).

(d) CENP-C$^{AID-EYFP/AID-EYFP}$ cells were subject to CENP-N depletion via siRNA, as in Fig. 7d, and assessed for centromeric CENP-T localization.

(e) Quantitation of centromeric CENP-T intensity. As expected, CENP-T levels are markedly reduced in the presence of CENP-N depletion[3,8,9], which indicates the expected impact of substantial CENP-N depletion by the siRNA approach used here and in the experiment in Fig. 7d-f. Mean ± 95% confidence interval (n>2000 centromeres in all cases).

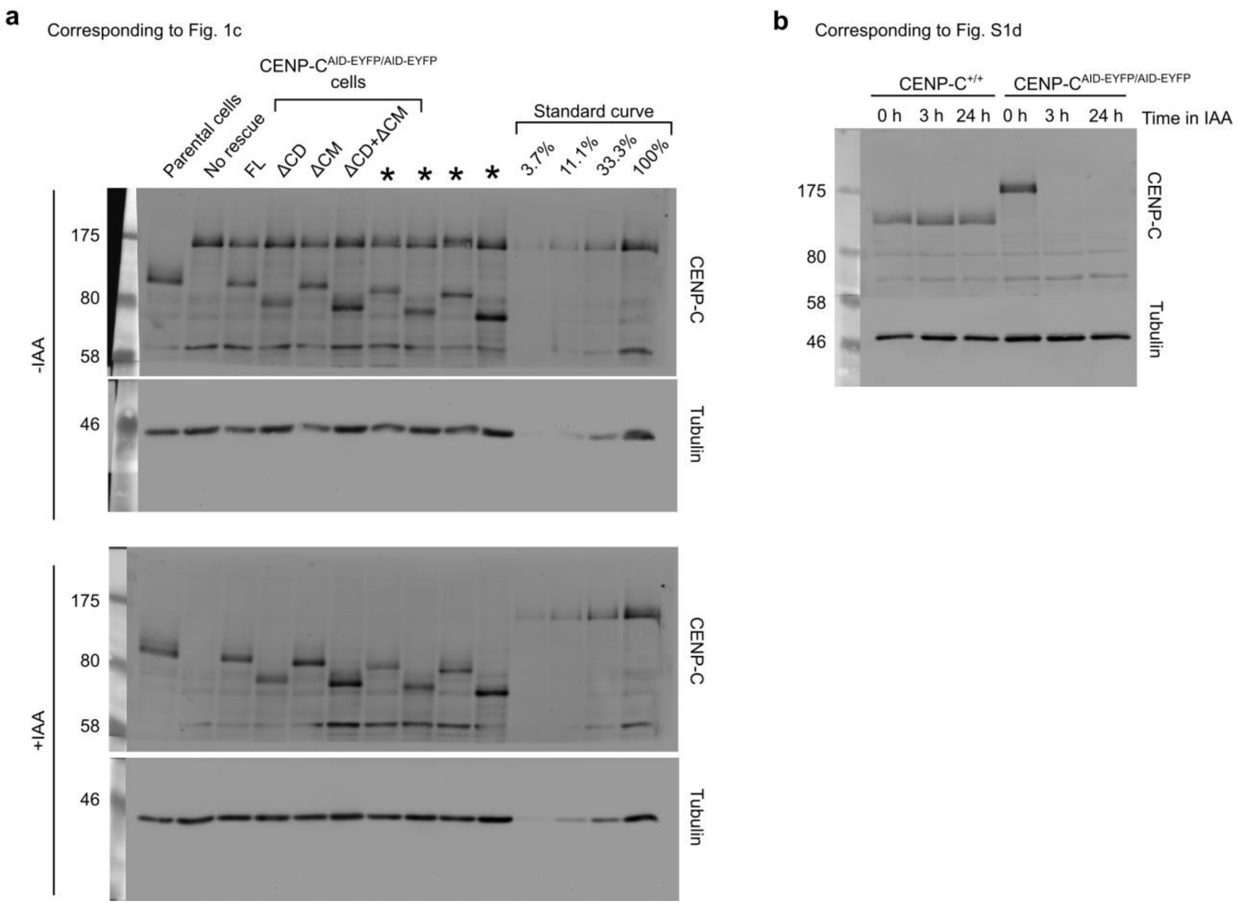

Supplementary Figure 9. Uncropped western blots.

(a-b) Uncropped blots corresponding to Fig 1c (a) and Fig. S1d (b). Membranes were cut prior to incubation with the indicated primary antibodies. Note that the western blot in Fig. S1j was not cropped in the first place, so it is not included in this supplementary figure.

SUPPLEMENTAL REFERENCES

1. Falk, S. J. *et al.* CENP-C reshapes and stabilizes CENP-A nucleosomes at the centromere. *Science* **348,** 699–704 (2015).

2. Cumming, G., Fidler, F. & Vaux, D. L. Error bars in experimental biology. *J. Cell Biol.* **177,** 7–11 (2007).

3. McKinley, K. L. *et al.* The CENP-L-N complex forms a critical node in an integrated meshwork of interactions at the centromere-kinetochore interface. *Mol. Cell* **60,** 886–898 (2015).

4. Walters, B. T., Ricciuti, A., Mayne, L. & Englander, S. W. Minimizing back exchange in the hydrogen exchange-mass spectrometry experiment. *J. Am. Soc. Mass Spectrom.* **23,** 2132–9 (2012).

5. Sekulic, N. & Black, B. E. A reader for centromeric chromatin. *Nat. Cell Biol.* **11,** 793–795 (2009).

6. Tachiwana, H. *et al.* Crystal structure of the human centromeric nucleosome containing CENP-A. *Nature* **476,** 232–235 (2011).

7. Black, B. E., Brock, M. A., Bedard, S., Woods, V. L. & Cleveland, D. W. An epigenetic mark generated by the incorporation of CENP-A into centromeric nucleosomes. *Proc. Natl. Acad. Sci. U. S. A.* **104,** 5008–5013 (2007).

8. Logsdon, G. A. *et al.* Both tails and the centromere targeting domain of CENP-A are required for centromere establishment. *J. Cell Biol.* **208,** 521–531 (2015).

9. Samejima, I. *et al.* Whole-proteome genetic analysis of dependencies in assembly of a vertebrate kinetochore. *J. Cell Biol.* **211,** 1141–1156 (2015).
